# Comparison of 3 T and 1.5 T for T2* magnetic resonance of tissue iron

**DOI:** 10.1186/s12968-016-0259-9

**Published:** 2016-07-08

**Authors:** Mohammed H. Alam, Dominique Auger, Laura-Ann McGill, Gillian C. Smith, Taigang He, Cemil Izgi, A. John Baksi, Rick Wage, Peter Drivas, David N. Firmin, Dudley J. Pennell

**Affiliations:** NIHR Cardiovascular Biomedical Research Unit, Royal Brompton and Harefield NHS Foundation Trust, London, UK; Imperial College, London, UK; St George’s Hospital, London, UK; Royal Brompton Hospital, Sydney Street, London, SW3 6NP UK

**Keywords:** Magnetic resonance, 3 T, Heart, Liver, Iron overload, Siderosis, T2*

## Abstract

**Background:**

T2* magnetic resonance of tissue iron concentration has improved the outcome of transfusion dependant anaemia patients. Clinical evaluation is performed at 1.5 T but scanners operating at 3 T are increasing in numbers. There is a paucity of data on the relative merits of iron quantification at 3 T vs 1.5 T.

**Methods:**

A total of 104 transfusion dependent anaemia patients and 20 normal volunteers were prospectively recruited to undergo cardiac and liver T2* assessment at both 1.5 T and 3 T. Intra-observer, inter-observer and inter-study reproducibility analysis were performed on 20 randomly selected patients for cardiac and liver T2*.

**Results:**

Association between heart and liver T2* at 1.5 T and 3 T was non-linear with good fit (*R*^2^ = 0.954, *p* < 0.001 for heart white-blood (WB) imaging; *R*^2^ = 0.931, *p* < 0.001 for heart black-blood (BB) imaging; *R*^2^ = 0.993, *p* < 0.001 for liver imaging). R2* approximately doubled between 1.5 T and 3 T with linear fits for both heart and liver (94, 94 and 105 % respectively). Coefficients of variation for intra- and inter-observer reproducibility, as well as inter-study reproducibility trended to be less good at 3 T (3.5 to 6.5 %) than at 1.5 T (1.4 to 5.7 %) for both heart and liver T2*. Artefact scores for the heart were significantly worse with the 3 T BB sequence (median 4, IQR 2–5) compared with the 1.5 T BB sequence (4 [3–5], *p* = 0.007).

**Conclusion:**

Heart and liver T2* and R2* at 3 T show close association with 1.5 T values, but there were more artefacts at 3 T and trends to lower reproducibility causing difficulty in quantifying low T2* values with high tissue iron. Therefore T2* imaging at 1.5 T remains the gold standard for clinical practice. However, in centres where only 3 T is available, equivalent values at 1.5 T may be approximated by halving the 3 T tissue R2* with subsequent conversion to T2*.

## Background

The transfusion dependent anemias are an important worldwide cause of morbidity and mortality. In these patients, repeated blood transfusions can lead to substantial iron tissue deposition which results in heart failure, endocrine dysfunction and death [1]. Iron chelation therapy is the crucial element of tissue siderosis prevention and treatment. However, the existing drug regimens vary in their efficacy, side-effects and potential toxicity. Therefore, tailored chelation therapy administration is mandatory and requires careful cardiac and liver iron content monitoring.

A number of studies have assessed the robustness, reliability and reproducibility of myocardial and liver iron content quantification by T2* magnetic resonance (MR) [2–5]. Myocardial T2* has been used extensively to monitor iron concentration in thalassemia patients [6]. The majority of these studies have described iron content assessment and its validation against tissue biopsy at 1.5 Tesla (T). However, 3 T scanners are increasingly being used for clinical indications, and may have some potential advantages, including higher signal-to-noise ratio and shorter acquisition times. Set against these advantages are issues of increased inhomogeneity in the magnetic fields of the magnet (B0 and B1), increased specific absorption rate, increased susceptibility artefacts, and T2* shortening [7]. There is rather limited data about reproducibility and the comparative robustness of myocardial and liver T2* and R2* at 3 T [8–11]. The purpose of this study was therefore to relate T2* and R2* at 3 T vs 1.5 T in a substantial sample size over a wide range of tissue iron concentrations, with evaluation of image quality, and reproducibility.

## Methods

### Patients and study design

A total of 104 consecutive subjects were prospectively recruited from referrals for siderosis screening. Furthermore, a group of 20 healthy volunteers was included. Patients were already scheduled to undergo T2* MR myocardial and liver content assessment at 1.5 T for clinical purposes and a research scan at 3 T was performed in addition. Inclusion criteria were: 1) age >18 years old and 2) written and informed consent to participate. Exclusion criteria were: 1) claustrophobia, 2) metallic implants or permanent pacemaker and 3) inability to hold recumbent position for >15 min. The study protocol was approved by North-East Thames ethics committee.

### Image acquisition

The T2* MR protocol consisted of two parts: 1) Myocardial and liver iron content assessment on a 1.5 T Sonata scanner 2) Repeated myocardial and liver iron burden assessment with a 3 T Skyra scanner (both Siemens Medical Systems, Erlangen, Germany).

#### Cardiac iron content

At 1.5 T, a four-element cardiac phase-array coil was used. After routine localizer acquisitions, ECG gated single breath hold multi-echo white blood (WB) and black blood (BB) sequences were acquired at a single mid-ventricular short axis slice with a 10 mm thickness at eight separate echo times (2.6–16.74 ms, at 2.02 ms increments). Both WB and BB sequences used a flip angle of 35°, a matrix of 128 × 256 pixels, a field of view (FOV) of 40 cm, repetition time of 20 ms between each radiofrequency pulse and a sampling bandwidth of 810 Hz per pixel. For WB imaging, all echo times were acquired exactly after the ECG R wave trigger. For BB sequence acquisition, the double inversion pulses were applied at the R wave trigger and the inversion time was set to extend into diastole.

At 3 T, similar sequences were run on each patient. For WB iron content assessment, echo time ranged from 2.69 ms to 18.86 ms with 2.31 ms increments with a matrix of 256 × 256 pixels and a FOV of 40 cm. For the BB acquisition, echo times ranges from 1.57 ms to 17.74 ms with similar time increments, matrix size and FOV size. An acceleration factor of 2 was applied on all sequences. A small shimming box was used over the heart to optimize the gradients settings and minimize artefacts.

#### Liver iron content

At 1.5 T, a single transaxial 10 mm thick slice through the center of the liver was acquired at a succession of 20 echo times ranging from 0.97 ms to 13.89 ms with 0.68 ms increments, using a non-ECG-gated gradient echo sequence with a flip angle of 20°, a matrix of 128 × 128 pixels a 40 cm FOV and a sampling bandwidth 1950 Hz per pixel. The TR between 2 RF pulses was 200 ms. A four channel-channel array coil was used.

At 3 T, sequence parameters were comparable. Echo time ranged from 1.0 ms to 16.5 ms with 1.41 ms increments. Matrix size was 128 × 128 pixels. Acceleration factor two was applied on all acquisitions. As for cardiac T2* and R2*, a small shimming box was used over the liver to improve image acquisition quality.

### Iron content quantification

The analysis of myocardial and liver T2* has been described elsewhere [4, 12]. In brief, dedicated software (Thalassaemia tools, a plug-in of CMRtools, Cardiovascular Imaging Solutions, London) was used. The entire thickness of the cardiac interventricular septum and a large region of interest within the liver parenchyma without blood vessels were selected to measure signal intensity (SI) at each echo time. The SI was plotted against echo time and a mono-exponential trendline was fitted to the decay curve to derive T2* according to this equation:

$$ SI=S{I_0}^{.}{e}^{{\textstyle \mathit{\hbox{-}}}TE/T2\ast } $$

T2* was subsequently transformed into its reciprocal R2* according to the equation:

$$ T2*=1000/R2* $$

Curve fitting was done according to the truncation method [13]. In patients with cardiac T2* < 10 ms or liver T2* < 3.3 ms at 1.5 T, a second-moment noise-corrected model was used [14]. Cardiac T2* image quality was prospectively evaluated for each acquisition according to the following scoring scale: 0-unusable: uninterpretable images, 1-poor: heart just discernible, 2-average: severe septal artefact, 3-good: moderate septal artefact, 4-very good: mild septal artefact: 5-excellent: no septal artefact. Higher scores therefore indicated less artefact and higher image quality.

### Reproducibility

In order to compare reproducibility at 1.5 T and at 3 T, a group of 20 patients was selected for inter-observer, intra-observer variability and for inter-study variability. The 20 selected patients had levels of myocardial and liver siderosis that covered the entire range of the disease (from absent to severe iron overload). For intra-observer variability, the same investigator was asked to reassess myocardial iron content on WB and BB sequences and liver iron at 1.5 T and 3 T within a month after the first scan. For inter-observer variability two investigators experienced in MR iron content evaluation separately reported T2* at each sequence at 1.5 T and 3 T for each selected patient. Finally, patients underwent a repeated 3 T cardiac and liver iron content evaluation after a period of 1 h after the previous scan, for inter-study reproducibility assessment.

### Statistical analysis

Imaging data were not normally distributed and are therefore expressed as median (interquartile range). Comparison of T2* and R2* values between normal volunteers and iron overload patients was performed with Mann-Whitney *U*-test. Association between T2* and R2* values according to different sequence acquisition and field strengths were evaluated with the Spearman’s correlation coefficient with 95 % confidence interval (CI) or non-linear regression when appropriate. Cardiac artefact scoring between different acquisitions and field strengths were compared using a Chi-Square test. Coefficients of variation (CoV) and intra-class correlation (ICC) (with 95 % CI) analysis were conducted to assess inter- and intra-observer variability in addition to inter-study variability of cardiac and liver T2* values at 1.5 T and 3 T. For ICC, an alpha two-way mixed model with absolute agreement analysis was used. Statistical comparison between BB sequence inter-and intra-observer and inter-study reproducibility at 1.5 T and at 3 T was performed using the squared difference between the 2 observations as an estimate of between subjects variance and was multiplied by 2. A paired *T*-Test was done to compare the different squared difference. When appropriate, the squared differences were log transformed to allow *T*-test use. When the difference was zero, it was replaced by half of the next value before log transformation [15]. Finally, Bland-Altman plots were generated to further describe the reproducibility of T2* measurements at 3 T [16]. A *P*-value <0.05 was considered significant. All analyses were done using SPSS software version 22.0.0, IBM, Chicago, Illinois, USA.

## Results

### Patient characteristics and comparison with healthy volunteers

A total of 124 subjects were included in the analysis. Demographics, clinical characteristics and median heart and liver T2* measurement at 1.5 T and at 3 T in both iron overload (*n* = 104) and normal (*n* = 20) patients are shown in Table 1. β-thalassemia major was present in 45 %. In the patients with iron overload, median age was 30 (23–53) years old. The majority of patients (73 %) were treated with at least one iron chelating agent. There were 7 patients with severe cardiac iron overload (cardiac T2* < 10 ms at 1.5 T), and 45 patients with severe liver iron overload (liver T2* < 3.3 ms at 1.5 T). Liver acquisitions were not performed in 4 patients due to a temporary scanner fault not related to the research.

**Table 1:** Patient characteristics

Variables	Patients	Controls	*P*-value
	*N* = 104	*N* = 20	
Age (years)	30 [23–53]	35 [26–43]	0.41
Gender (Male/Female)	55/45	10/10	0.67
Diagnosis			
TM	47		
SCA	15		
Other hemoglobinopathy	20		
MDS	4		
Other	18		
1.5 T WB Heart T2*	30.3 [22.6–36.8]	32.3 [28.9–36.7]	0.20
3 T WB Heart T2*	18.5 [11.8–24.3]	20.5 [18.3–24.3]	0.14
1.5 T Liver T2*	4.2 [1.8–8.6]	25.8 [23.1–28.0]	<0.001
3 T Liver T2*	2.3 [0.9–4.7]	17.3 [14.8–21.4]	<0.001

Data are expressed as median [interquartile range]. *SCA* sickle cell anemia, *MDS* myelodysplasia syndrome, *TM* thalassemia major, *WB* white blood

### Association between cardiac T2* and R2* at 1.5 T and 3 T

**Fig. 1:**
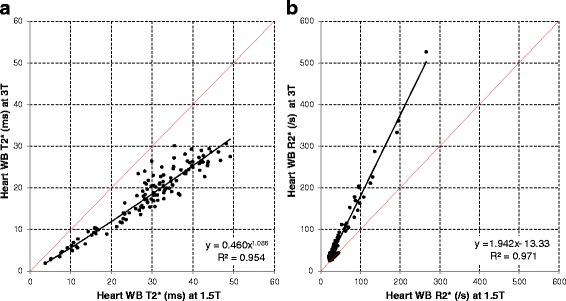
**a** Association between cardiac T2* measurements at 1.5 T and 3 T using the white- blood sequence. The *red line* is the line of identity, and the *black line* is the best fit regression line. **b** Association between cardiac R2* measurements at 1.5 T and 3 T using the white-blood sequence. The *red line* is the line of identity, and the *black line* is the best fit regression line

**Fig. 2:**
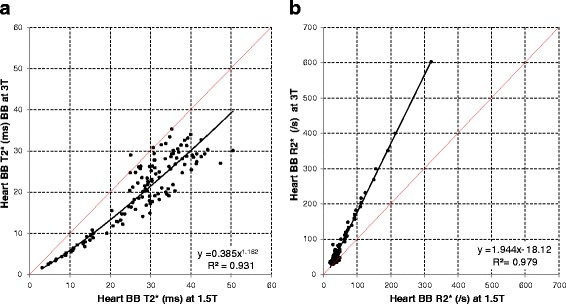
**a** Association between cardiac T2* measurements at 1.5 T and at 3 T using the black-blood sequence. The *red line* is the line of identity, and the *black line* is the best fit regression line. **b** Association between cardiac R2* measurements at 1.5 T and 3 T using the black-blood sequence. The *red line* is the line of identity, and the *black line* is the best fit regression line

Using the WB sequence, the relationship between T2* values at 1.5 T and 3 T for the heart was non-linear with good fit (*R*^2^ = 0.954, *P* < 0.001) (Fig. 1a). There was good linear correlation between R2* values at 1.5 T and at 3 T with the WB sequence (*R*^2^ = 0.971, *P* < 0.001) (Fig. 1b) with near doubling (94 %) of R2* values at 1.5 T. Association between T2* and R2* values at 1.5 T and 3 T using the BB sequence are displayed in Fig. 2a and b. The relationship between BB T2* values at 1.5 T and 3 T was non-linear with good fit (*R*^2^ = 0.931, *P* < 0.001). Good linear correlation was found for BB R2* measurements at 1.5 T and at 3 T (*R*^2^ = 0.979, *P* < 0.001) again with near doubling (94 %) of R2* values at 1.5 T.

### Association between liver T2* and R2* at 1.5 T and at 3 T

**Fig. 3:**
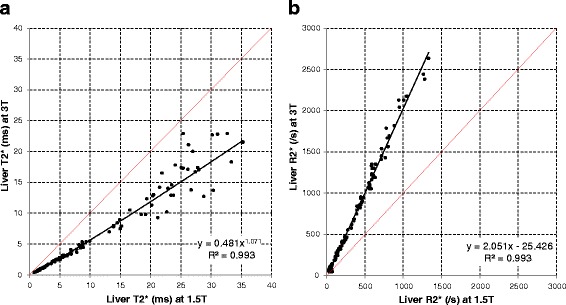
**a** Association between liver T2* measurements at 1.5 T and at 3 T. The *red line* is the line of identity, and the *black line* is the best fit regression line. **b** Association between liver R2* measurements at 1.5 T and at 3 T. The *red line* is the line of identity, and the *black line* is the best fit regression line

Regression graphs between T2* and R2* at 1.5 T and 3 T for the liver are displayed in Fig. 3a and b. For T2*, an excellent non-linear fit was found between the values obtained at 1.5 T and at 3 T (*R*^2^ = 0.993, *P* < 0.001). Similar to heart R2* values, excellent linear correlation was found between R2* values at the two different field strengths (*R*^2^ = 0.993, *P* < 0.001) with an increase of 105 % in R2* from 1.5 T to 3 T.

### Artefact scores

**Fig. 4:**
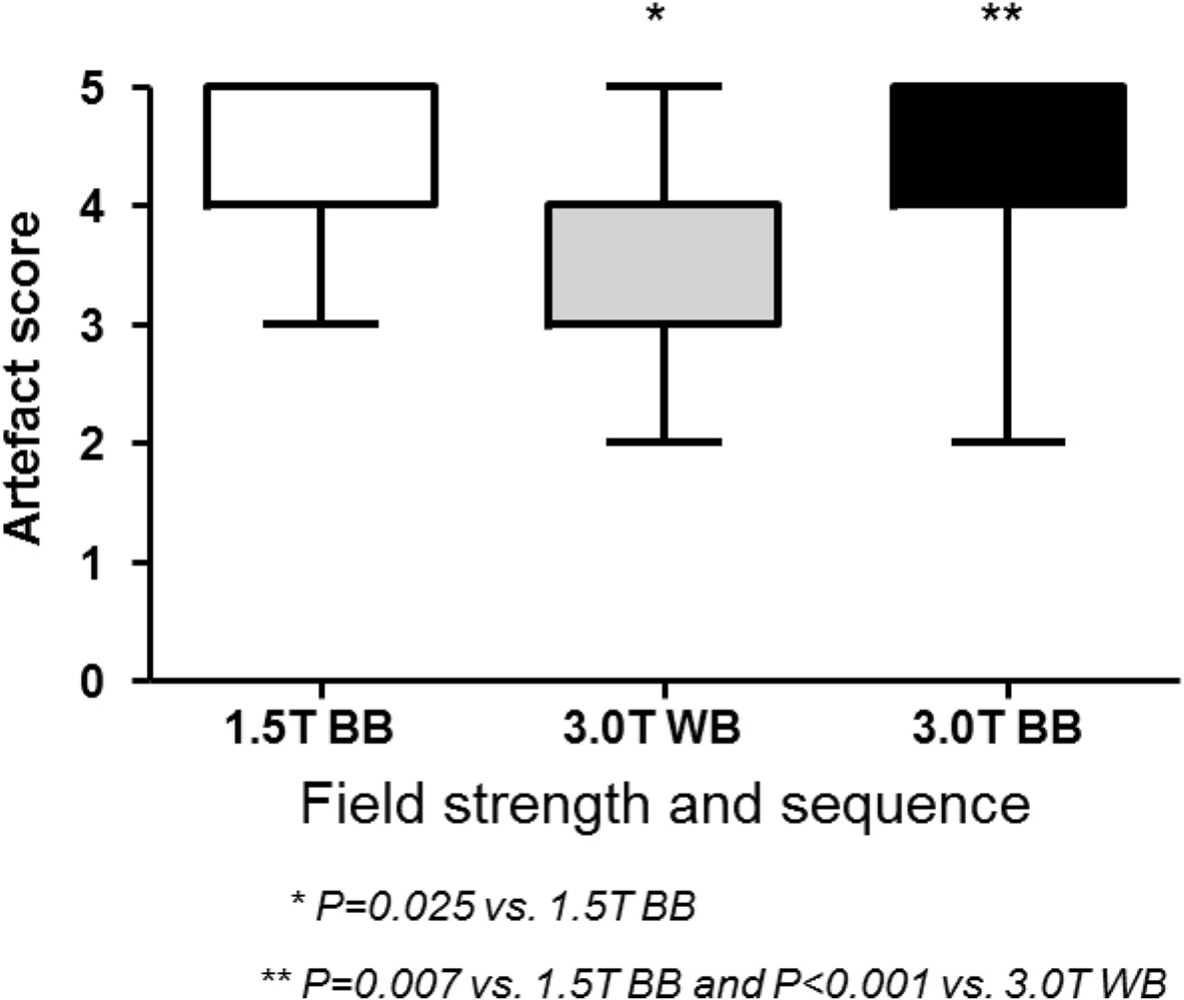
Comparison of artefacts scores for cardiac iron content assessment according to the field strength and the type of sequence used for image acquisition

Median artifact score was 4 (4–5) with the 1.5 T BB sequence, 4 (3–4) with the 3 T WB sequence and 4 (4–5) with the 3 T BB sequence. The artifact score was significantly higher (higher indicates less artefact) with the BB sequence at 1.5 T than with the WB sequence at 3 T (*P* = 0.025) and the BB sequence at 3 T (*P* = 0.007). Moreover, the artefact score was significantly superior with the BB sequence at 3 T than with the WB sequence at 3 T (*P* < 0.001) (Fig. 4).

### Reproducibility

**Fig. 5:**
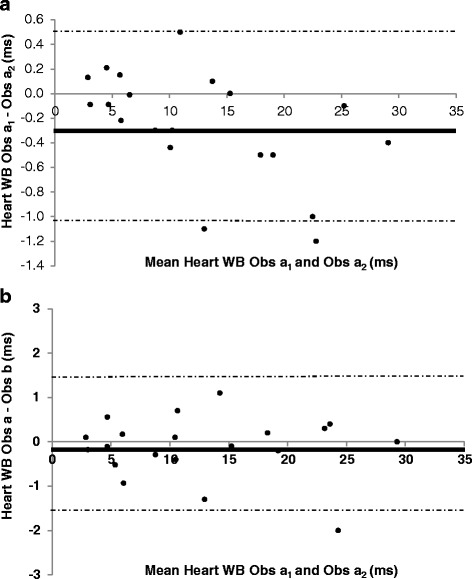
Bland-Altman plots for intra-observer variability (Panel **a**) and inter-observer variability (Panel **b**) of White-Blood T2* measurements at 3 T

**Fig. 6:**
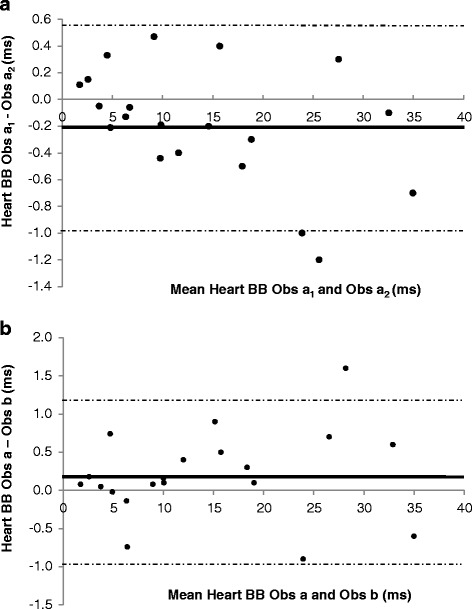
Bland-Altman plots for intra-observer variability (Panel **a**) and inter-observer variability (Panel **b**) of Black-Blood T2* measurements at 3 T

**Fig. 7:**
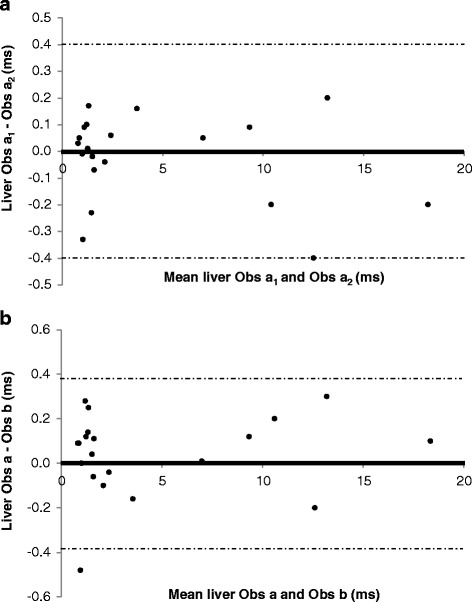
Bland-Altman plots for intra-observer variability (Panel **a**) and inter-observer variability (Panel **b**) of Liver T2* measurements at 3 T

The evaluation of T2* intra-observer variability is shown in Table 2. There was excellent agreement between the repeated measurements in all sequences at both 1.5 and 3 T, with mildly increased CoV (inferior reproducibility) at 3 T compared to 1.5 T. The Bland-Altman plots confirm that there was no systemic bias in repeated assessment of T2* at 3 T by the same observer (Figs. 5a, 6a, 7a).

**Table 2:** Coefficients of variation and intra-class correlation for intra-observer reproducibility of T2* measurements according to acquisition sequence and magnetic field strength

	Heart				Liver	
	WB		BB			
	CoV	ICC (95 % CI)	CoV	ICC (95 % CI)	CoV	ICC (95 % CI)
1.5 T	3.19 %	0.999 (0.998–1.000)	1.54 %	1.000 (0.999–1.000)	1.98 %	0.953 (0.890–0.980)
3 T	3.54 %	0.999 (0.997–1.000)	3.62 %	0.999 (0.999–1.000)	3.62 %	0.999 (0.997–1.000)

*BB* Black Blood, *CI* Confidence Interval, *CoV* Coefficient of Variation, *ICC* Intra-class Correlation, *WB* White Blood

Table 3 displays the CoV and ICC for estimation of T2* inter-observer variability. There was excellent agreement between the two observers. Bland-Altman plots confirms the agreement between the observers at 3 T (Figs. 5b, 6b and 7b) without the presence of systematic bias. Finally, inter-study reproducibility was excellent as shown in Table 4. CoV was found to be slightly inferior at 3 T than at 1.5 T. Overall, at 3 T, the BB imaging sequence had less variability of cardiac T2* measurements than the WB sequence but this did not achieve statistical significance (Table 5).

**Table 3:** Coefficients of variation and intra-class correlation for inter-observer reproducibility of T2* measurements according to acquisition sequence and magnetic field strength

	Heart				Liver	
	WB		BB			
	CoV	ICC (95 % CI)	CoV	ICC (95 % CI)	CoV	ICC (95 % CI)
1.5 T	3.34 %	0.999 (0.998–1.000)	2.76 %	0.999 (0.999–1.000)	4.24 %	1.000 (0.999–1.000)
3 T	5.50 %	0.998 (0.990–0.998)	4.01 %	0.999 (0.998–1.000)	3.96 %	0.999 (0.996–1.000)

*BB* Black Blood, *CI* Confidence Interval, *CoV* Coefficient of Variation, *ICC* Intra-class Correlation, *WB* White Blood

**Table 4:** Coefficients of variation and intra-class correlation for inter-study reproducibility of T2* measurements according to acquisition sequence and magnetic field strength

	Cardiac				Liver	
	WB		BB			
	CoV	ICC (95 % CI)	CoV	ICC (95 % CI)	CoV	ICC (95 % CI)
1.5 T	5.55 %	0.996 (0.989–0.998)	4.55 %	0.994 (0.985–0.998)	5.69 %	0.979 (0.949–0.992)
3 T	7.68 %	0.970 (0.926–0.988)	6.48 %	0.996 (0.990–0.998)	6.31 %	0.998 (0.993–0.999)

*BB* Black Blood, *CI* Confidence Interval, *CoV* Coefficient of Variation, *ICC* Intra-class Correlation, *WB* White Blood

**Table 5:** Reproducibility of Black Blood T2* at 1.5 T and 3 T

Test	Geometric Mean (95 % CI) of Squared difference		*P*-value
	T2* BB 1.5 T	T2* BB 3 T	
Intra-Observer	0.04 (0.02, 0.09)	0.07 (0.03, 0.15)	0.26
Inter-Observer	0.04 (0.01, 0.12)	0.07 (0.02, 0.21)	0.18
Inter study	0.26 (0.08, 0.80)	0.28 (0.09, 0.82)	0.94

## Discussion

The main findings of the study are: a) T2* shortening and increased B0 and B1 inhomogeneities may make T2* quantification at 3 T difficult in high iron loading; b) The correlations between T2* and R2* values between 1.5 T and 3 T are high; c) Good image quality was achieved at 3 T but was associated with significantly more artefacts than conventional BB 1.5 T imaging; d) There was a trend towards T2* measurements at 3 T being less reproducible than at 1.5 T.

To our knowledge, this is the largest study of transfusion dependent patients reporting the association between T2* and R2* values at 1.5 T and 3 T. Meloni reported on 38 patients transfusion dependent patients and showed good correlation between WB mid-ventricular septal cardiac R2*at 1.5 T and 3 T (*R*^2^ = 0.934) [8]. The authors also reported on the correlation between liver R2* at 3 T vs 1.5 T. Unfortunately, in their study the data from five patients with high iron overload (high R2*) had to be excluded to respect the best-fit correlation analysis. The present study includes all 45 patients with severe liver iron overload at 1.5 T and used a second order truncation method to obtain reliable T2* values at 3 T in all these patients, with good associations between 1.5 T and 3 T values [14].

Guo quantified liver and cardiac T2 and T2* at 3 T in 24 patients and eight normal subjects [9]. The T2* value could not be reliably be determined using their BB 3 T T2* protocol in certain severely iron overloaded patients with heart or liver T2* <2 ms (heart *n* = 6; liver *n* = 8). Thus, reliable 3 T T2* values in high-risk patients were not always obtainable. Storey also expressed concerns over T2* and R2* quantification at 3 T [10]. Indeed, inadequate shimming and the ultrashort echo times needed to appropriately assess very low T2* at 3 T may produce inaccuracies in T2* quantification. Moreover, the increase in RF power deposition at 3 T limits the capacity to increase RF gradients. Our study also showed that cardiac 3 T T2* acquisition is associated with poorer image quality (artefact scores lower) for both WB and BB which resulted in less good reproducibility at 3 T than at 1.5 T. This important issue may hamper patient care and therefore T2* assessment at 1.5 T remains the clinical test of choice. At centres where only 3 T scanners are available, then it would be reasonable to convert the 3 T value for T2* to a value that approximates to what would be expected at 1.5 T, but safeguards must be in place to ensure that the T2* value at 3 T has been obtained without compromise related to artefact and analysis. This is best performed by halving the R2* value at 3 T and divide into 1000 to estimate the T2* equivalent at 1.5 T. Another possible approach to assessing iron loading at 3 T is to use native T1 mapping, but this needs further research [17].

### Limitations

The present study was conducted in a single centre with experience in T2* assessment. The relatively low CoV and high ICC values obtained should ideally be compared to the reproducibility data from other centres. One advantage conferred by 3 T MR may not have been fully explored in the present study, which is the possible increase of the acceleration factor and consequent shortening of the acquisition time. Such an increase in image acquisition speed however, may lead to reduction in signal to noise ratio and loss of image quality. Only adults >18 years were studied in this research which may limit the conclusions that may be drawn in younger and smaller patients.

## Conclusion

In transfusion dependent anaemia patients, heart and liver T2* and R2* quantification is feasible at 3 T. Good associations were obtained between values at 3 T and at 1.5 T with good reproducibility at both field strengths. However, no clear advantage of T2* imaging at 3 T could be identified and the clinical routine of T2* MR being performed at 1.5 T remains clinically optimal and should continue. Where only 3 T is available, one option for iron quantification is to halve the tissue R2* found at 3 T, and divide into 1000  to estimate the equivalent T2* value which would have been found at 1.5 T. However, great care must be taken to ensure that the T2* value at 3 T is not compromised by artefact or analysis difficulty.

## Abbreviations

BB, black blood; CMR, cardiovascular magnetic resonance; CoV, coefficient of variation; FoV, field of view; ICC, intraclass correlation coefficient; ROI, region of interest; T, Tesla; WB, white blood
